# A Computational Study of a Recreated G Protein-GEF Reaction Intermediate Competent for Nucleotide Exchange: Fate of the Mg Ion

**DOI:** 10.1371/journal.pone.0009142

**Published:** 2010-02-18

**Authors:** Mériam Ben Hamida-Rebaï, Charles H. Robert

**Affiliations:** CNRS Institute of Biochemistry and Molecular and Cellular Biology (IBBMC), Université Paris-Sud 11, Orsay, France; German Cancer Research Center, Germany

## Abstract

Small G-proteins of the superfamily Ras function as molecular switches, interacting with different cellular partners according to their activation state. G-protein activation involves the dissociation of bound GDP and its replacement by GTP, in an exchange reaction that is accelerated and regulated in the cell by guanine-nucleotide exchange factors (GEFs). Large conformational changes accompany the exchange reaction, and our understanding of the mechanism is correspondingly incomplete. However, much knowledge has been derived from structural studies of blocked or inactive mutant GEFs, which presumably closely represent intermediates in the exchange reaction and yet which are by design incompetent for carrying out the nucleotide exchange reaction. In this study we have used comparative modelling to recreate an exchange-competent form of a late, pre-GDP-ejection intermediate species in Arf1, a well-characterized small G-protein. We extensively characterized three distinct models of this intermediate using molecular dynamics simulations, allowing us to address ambiguities related to the mutant structural studies. We observed in particular the unfavorable nature of Mg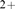 associated forms of the complex and the establishment of closer Arf1-GEF contacts in its absence. The results of this study shed light on GEF-mediated activation of this small G protein and on predicting the fate of the Mg ion at a critical point in the exchange reaction. The structural models themselves furnish additional targets for interfacial inhibitor design, a promising direction for exploring potentially druggable targets with high biological specificity.

## Introduction

Small G-proteins of the Ras superfamily [1] are single-subunit proteins functioning as molecular switches. They can be maintained in an activated or deactivated state according to the presence of bound GTP or GDP, respectively [2]. While deactivation occurs by regulated hydrolysis of the GTP phosphodiester bond, activation requires dissociation of the resulting GDP molecule, followed by binding of a new GTP. Spontaneous GDP dissociation is slow (on the order of hours [3]), so nucleotide exchange in the cell is accelerated and regulated by guanine-nucleotide exchange factors, or GEFs. The GEF binds to the inactive G-protein-GDP complex and facilitates expulsion of the GDP. Understanding this reaction in detail is the goal of much current research. Several human diseases involve G-protein up regulation [4]. Specific inhibition of GEF action has thus attracted significant attention, with the G-protein-GEF interface itself as a potential therapeutic target [3], [5]–[8].

The Arf family of small G proteins are involved in vesicular protein trafficking within the cell. They were discovered as a consequence of their role in cholera infection [9], and have since been implicated in several human disease processes including HIV infectivity [10], pathogenic bacterial infection [11], and breast cancer proliferation [12]. Arf GEFs are characterized by their Sec7 domain in which the catalytic activity resides [13]. In the multiple alignment of 52 Sec7-domain proteins, Cox et al. [14] observed a high correspondence between known Sec7 structural features and alignment breakpoints, suggesting that the principal structure-function determinants are shared throughout the entire Sec7 family of GEFs. Indeed, Arf-GEFs from yeast can be used to catalyze nucleotide exchange in human Arfs [15]. Arf1 in particular has yielded particularly rich structural information in the form of crystal-structures obtained along the nucleotide exchange reaction pathway. These include the GDP-bound and GTP-bound Arf1 endpoints, free Sec7 domains, and several intermediates in which the Arf1-GEF complex was captured at different points in the exchange reaction by either point mutation or judiciously chosen conditions [6], [15]–[19]. These structural snapshots have furnished unprecedented insight into the overall steps involved in the exchange reaction.

GEF-promoted nucleotide exchange involves protein-protein complex formation with large structural rearrangements. The first half of the exchange reaction, *i.e.*, up to the dissociation of GDP, can be written schematically:
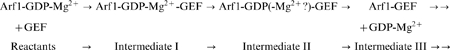



The intermediates indicated in this scheme are presumed to be closely represented by crystal structures, as summarized in Renault et al (2003) [6], which reveal several important conformational changes in the G-protein and the GEF. The initial binding of the GEF to inactive Arf1-Mg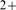-GDP is accompanied by closure of the exchange factor hydrophobic groove separating the N- and C-terminal subdomains [6], [19] and is accompanied by extraction of the beta strand of switch 1 from its pairing with the so-called “interswitch toggle”, resulting in intermediate I. The two partner proteins then associate more closely, accompanied by a shift of the interswitch along its axis, which blocks intramolecular binding of the myristoylated N-terminal helix and leads to intermediate II. This conformation favors membrane association of the complex and frees up space to accommodate the eventual P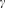 of the GTP [6]. Actual GDP dissociation is accomplished between intermediate forms II and III, the latter represented by the nucleotide-free intermediate resolved by Goldberg [15]. In this structure, at the midpoint of the exchange reaction, the interface with the GEF is more extensive and the P-loop has been transformed into another turn of the Arf1 helix 1.

While the crystal structure data provide a uniquely detailed view of the Sec7-mediated exchange reaction, fundamental mechanistic questions remain unanswered. For example, in GDP ejection it is presumed that a conserved Glutamate residue in the GEF acts on the phosphate moiety of the GDP by electrostatic repulsion, but details of this mechanism have not yet been obtained. Also, in most small G-proteins, some of the GDP binding energy comes from Mg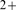, which at high concentration has been shown to have an inhibitory effect on GDP-GTP exchange [20]. Molecular dynamics simulations of GDP-bound but Mg-free G-proteins [21] led those authors to suggest that Mg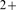 dissociation should occur in order for the GEF to bind. Yet crystal structures [6], [19] of the earliest available intermediate structure in the Arf system, that of the small-ligand BFA-inhibited Arf1-GDP-BFA-GEF complex, indicate that Mg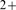 is still present. Thus Mg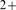 dissociation is not a prerequisite for initial GEF binding, and indeed its presence may be required [22]. The question of whether Mg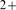 dissociation precedes GDP ejection remains open even after the structural resolution [6] of the complex formed between Arf1-GDP and the dominant-lethal GEF mutant E156K first identified by Beraud-Dufour et al. (1998) [23]. There is no sign of the Mg ion in this structure, which is consistent with the experimental observation that high [Mg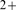] inhibits the formation of the mutant complex [23]. However, the positively-charged Lys sidechain, which replaces the catalytic Glu residue in this mutant, partially occludes the Mg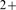 binding site near the diphosphates [6]. It is thus not possible to say whether the absence of Mg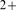 in this structure is an artifact of the inactivating Lysine substitution or if Mg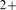 dissociation naturally occurs at this stage.

The heart of the nucleotide exchange reaction lies in the passage from intermediate II to intermediate III, during which GDP expulsion occurs. Yet as just mentioned our understanding of the pre-ejection intermediate species II is incomplete, being derived from an inactive mutant protein. This situation is not uncommon–intermediate species in a chemical or biological reaction are intrinsically difficult to study experimentally due to their low population and transience. This is where molecular modelling and simulations provide complementary tools for exploring the structural and dynamic properties of proteins and other biological macromolecules. Indeed, each distinct crystal structure in the Arf system offers a potential departure point for theoretical studies of the conformational dynamics of the corresponding complex in the vicinity of the reaction pathway. In the present study we have used molecular modelling to recreate a native-sequence, exchange-competent form of the late, pre-GDP-ejection intermediate II, based on the structure of the inactive mutant complex. We have extensively characterized three models of this intermediate using molecular dynamics simulations, shedding light in particular on Arf1-GEF interactions and the fate of the Mg ion in the exchange reaction. These results open the door to mechanistic studies of nucleotide ejection, at the heart of small G-protein activation. The structural models themselves furnish additional targets for interfacial inhibitor design [7], which has emerged as a promising direction for exploring potentially druggable targets with high biological specificity.

## Results

The guanine-nucleotide exchange reaction, like all reactions involving macromolecules, takes place in a conformational space of very high dimension. While the actual path taken by a particular G-protein-GEF complex through this enormous space cannot be predicted, it is likely that a common, more restrained region of the conformational space will envelope most such paths, particularly near the transition state. The structures of known G-protein-GEF complexes each constitute a point in this space, lying in or near this reaction path region. However, experimental structure determination frequently necessitates the use of devices such as modified substrates, inhibitors or inactivating mutants in order to trap intermediate structures. Such devices can introduce ambiguity in interpretions of the structural results, as a resulting complex may not lie sufficiently close to the reaction pathway to permit accurate mechanistic conclusions to be drawn.

To better understand GEF-mediated GDP dissociation, we used computational approaches to recreate a putative on-pathway Arf1-GDP-GEF species immediately preceding nucleotide ejection, indicated in the schema above as intermediate II. This complex was modelled on the structure of the abortive intermediate complex 1r8s of Renault et al [6], itself obtained by an inactivating GEF mutation E156K, by here reversing the mutated residue to the original “catalytic” Glu. In recreating this active complex, we had to confront the intrinsic ambiguity related to the absence of Mg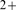 in the (inactive) 1r8s structure carrying the charge-reversal mutation. The first major possibility is that Mg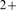 dissociated at a previous step, although necessarily after the formation of the initial complex I represented by the Arf-GDP-Mg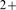-BFA-GEF complex (1s9d), in which this ion is present [6]. We refer to the resulting model for intermediate II as complex IIo, in which the “o” indicates the absence of Mg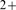. A second possibility is that the ion may have simply been displaced artefactually by the positively-charged amino group of the mutant lysine residue, in which case the Mg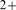 must be re-introduced into the model structure of the native complex. Indeed, an example of this line of reasoning is evident from the inclusion of Mg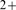 in Figure 4 of the article by Renault et al. [6]. In the current study, two flavors of the putative Mg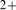-containing complex were considered. In the first, IIm1, the Mg ion was placed in the “canonical” position seen in inactive Arf1-GDP, in which coordination was made only with oxygen from the beta phosphate. An alternative placement, in the complex denoted IIm2, corresponds to the position of the sidechain amino group of the mutant Lys (see Methods). This placement allowed coordination by oxygen atoms from both alpha and beta phosphates. Such a coordination geometry has been observed in crystal structures and in MD simulation studies of ADP-Mg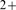 interactions [24].

**Figure 1:**
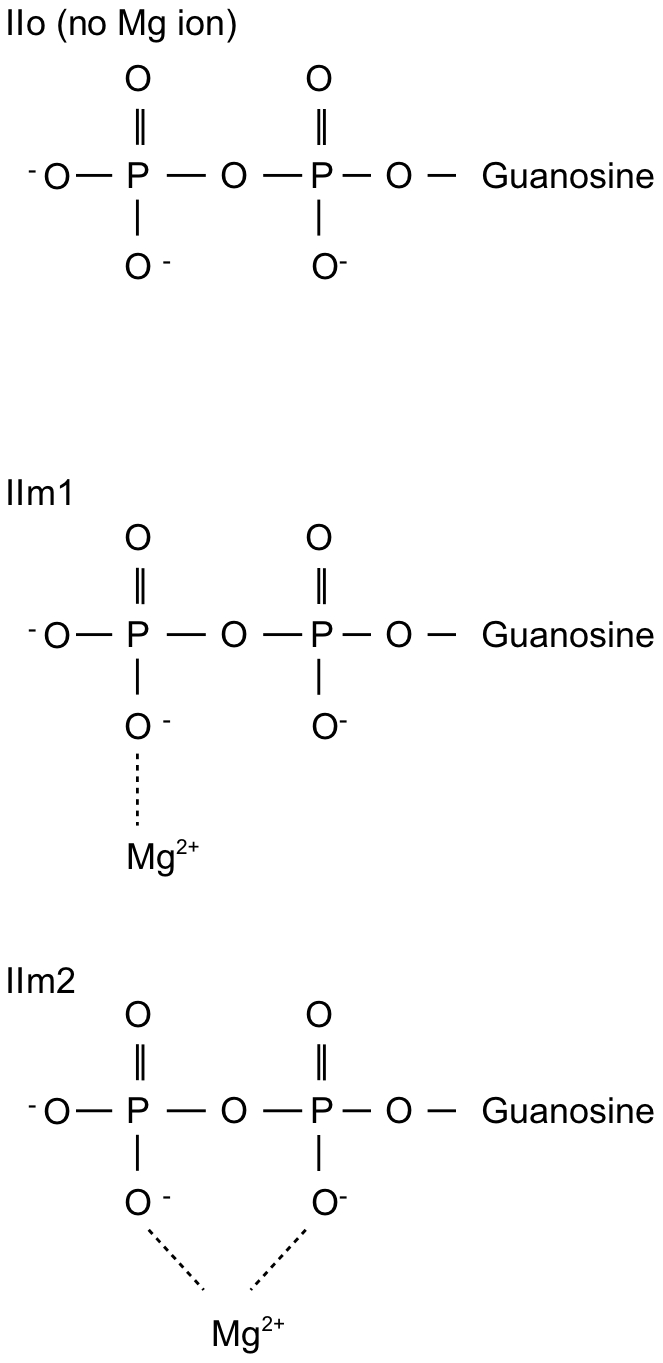
Mg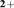 placement for the three starting models of the Arf1-GDP-GEF complex.

These three alternative models for the Arf1-GDP-GEF species II are represented schematically in Figure 1 and are summarized here:

- IIo: No Mg present
- IIm1: Mg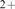 bound in the “canonical” position
- IIm2: Mg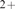 bound in the vicinity of the positively-charged sidechain amino group of the mutant Lys

Each of these reconstructed intermediate complexes was created using comparative modelling and characterized using molecular dynamics simulations.

### Conformational Stability of Reconstructed Intermediates

For each model, two MD runs were performed for a total of 30 ns of simulation time. The overall behavior of the different Arf1-GDP-GEF complexes was stable on the timescales of the simulations. This was seen by the C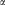 rms distance of the protein from the starting structure after best-fit superposition as a function of time, which was seen in all runs to stabilize after about 500 ps during the 1 ns equilibration period. Nevertheless, the conformational variation of the Arf1-GDP-GEF complexes differed significantly for the three different models, as summarized in the first column of Table 1. The Mg-free complex (IIo) showed the least deviation from the starting structure, with an rmsd of 1.15 A, while the values for the Mg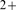-containing structures IIm1 and IIm2 were larger by 0.2 to 0.4 A, respectively, with correspondingly higher standard deviations.

**Table 1:** Conformational stability 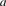 of intermediate Arf1-GDP-GEF complexes.

System	Complex 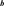	Arf1 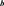	Arno 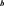	Arno 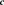
IIo	1.15 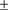 0.11	1.04 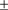 0.13	0.95 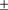 0.10	1.77 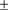 0.35
IIm1	1.33 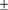 0.13	1.21 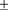 0.17	1.03 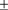 0.12	2.05 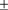 0.44
IIm2	1.55 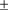 0.17	1.22 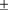 0.14	1.23 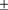 0.18	2.77 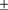 0.63


 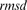, in 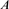.

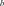
 after C  superposition of the indicated species.

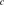
 after C  superposition on Arf1.

Calculating the rmsd after best-fit superimposition of the complex as a whole can mask deviations arising from domain and subdomain movements or fluctuations at a smaller scale. For this reason we also calculated the rmsd after superimposing the complex in different ways. Superimposing the complex on the separate components revealed each protein to be quite stable individually, as seen by the two central columns in Table 1. However, the rmsd statistics collected for Arno after superimposing on Arf1 showed much larger deviations, as seen in the last column of this Table. This value indicates the magnitude of orientational fluctuations of the two protein components with respect to each other in the complex. We note that, regardless of the superposition criteria employed, each of the measurements shown in Table 1 suggest the Mg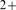-free form to be the most stable of the three models of intermediate II in terms of conformational variability.

### Mg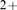 Interaction

**Figure 2:**
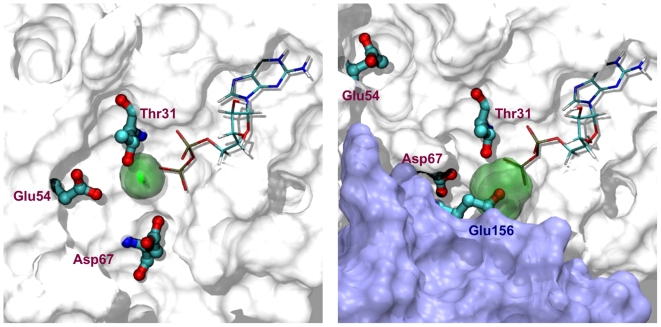
Mg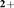 localization during MD trajectories after superposition on the GDP. *Left:* Arf1-GDP (1hur), *Right:* Arf1-GDP-GEF (model IIm2). Arf1 is shown in white. In the right panel the GEF is shown in light blue together with catalytic residue 156. Region of Mg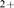 localization is shown as a transparent green surface.

In model species IIm1, the Mg ion proved to be well localized, with an rms fluctuation of 0.42 A in the coordinate system of the GDP, which is essentially identical to that seen in the reference Arf1-GDP simulation and in Ras MD studies [25]. In species IIm2 the Mg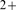 was far more mobile, with an rms fluctuation of 0.84 A (Figure 2). The Mg ion in this model is more closely associated with the sidechain of GEF residue Glu156, which as mentioned above is more mobile than in IIm1.

**Figure 3:**
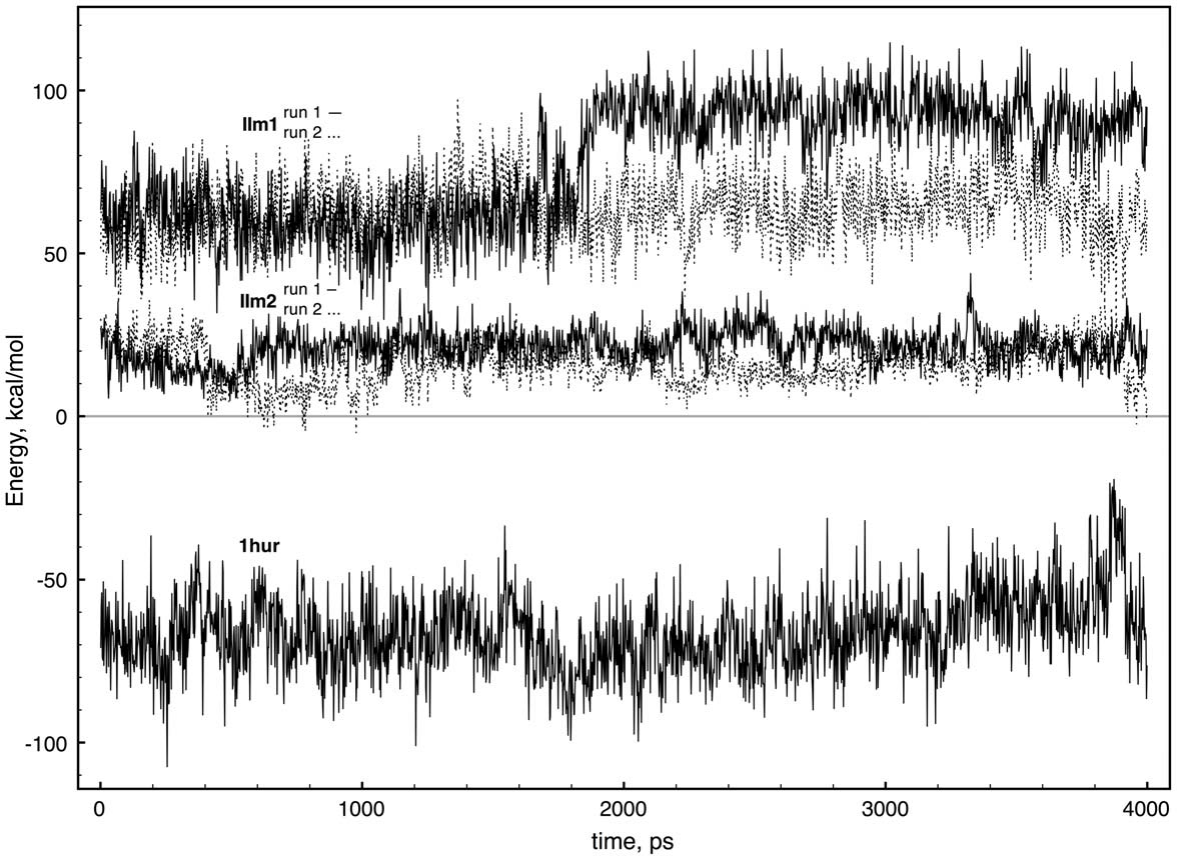
Energy of interaction between Arf1 and Mg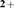. Interaction energy between Arf1 and Mg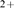 in the molecular dynamics simulations for the Mg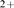-containing model complexes IIm1 and IIm2 (top and middle sets of lines, respectively) and for the reference Arf1-GDP-Mg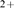 system (1hur) seen at bottom, presented as a function of simulation time.

Limited positional fluctuations do not necessarily reflect a strong interaction between Mg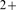 and Arf1. In Arf1-GDP alone, the energy of interaction measured between Arf1 and Mg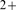 (Figure 3) was strongly negative; while in the intermediate complexes the interaction became somewhat (IIm2) or substantially (IIm1) positive. Two factors contribute to this effect. First, in the inactive Arf1-GDP complex, Arf1 residues Glu54 and Asp67 interact closely (carboxylate distances of 3.7 and 4.6 A, respectively) with the Mg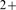. In the transformation to intermediate II, the so-called “interswitch” beta-hairpin containing both residues is displaced [6], as reflected in Figure 2, which modifies the respective distances to at least 16.0 and 3.8 A and thus removing a source of Arf1's electrostatic stabilization of the Mg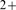. Second, in the inactive complex Arf1-GDP, the Mg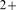 interacts with Arf residue Thr31 and with one of the GDP beta phosphate oxygens [26]. The Thr31OG-Mg distance remains on the order of 2.3 A in the inactive Arf1-GDP complex and in intermediate I of the exchange reaction. In both models IIm1 and IIm2, this interaction is modified. In IIm2, the interaction is lost due to the initial Mg ion placement in this system. In IIm1, in which the Mg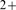 was initially placed in the position seen in the inactive Arf1-GDP complex, as well as in intermediate I, the Thr31OG distance was found to increase to 3.1 A as the Mg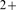 drew closer to the second beta-phosphate oxygen during the equilibration period. The ion thus gained electrostatic interaction with the GDP at the expense of its interaction with Arf1. The motor for this modification appears to be the approach of the GEF. In particular, the N-terminal of GEF helix 7, which could contribute via a helix dipole effect, is much closer to the Mg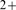 binding site (5 A) in intermediate II than in intermediate I (12 A) due to the maturing of the Arf1-GEF interface. Indeed, GEF residue Gln158 alone, at the N-terminal of this helix, was seen to add +5 kcal/mol to the interaction energy with the Mg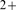.

The interaction energies given in Figure 3 must be interpreted with care: the standard molecular dynamics protocol used here does not reflect atom polarizability [27], [28]. Limitations of the present calculations are discussed separately (see Discussion). Nevertheless, the change of sign in the interaction energy for both models suggests marked weakening of the Arf1 interaction with Mg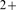 in intermediate species II.

### Arf1-GEF and GDP-GEF Interactions

The surface buried in the Arf1-GEF interface was followed throughout the simulations in order to identify potential modifications of the protein-protein interface. The results for the three models are presented in the first column of Table 2. The area of the Arf1-GEF interface for the three models was more than 3000 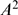, and falls in the range characterizing large biological interfaces [29]. A dominant contribution to this interface area is the burial of switch1 in the GEF hydrophobic groove. The interface was seen to be larger by more than 200 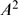 in the absence of Mg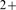 than in its presence. The more extensive interface is consistent with the analysis of the conformational fluctuations of the Arf1-GEF complexes presented above, with the larger interface associated with the “tighter”, or less fluctuating, complex.

**Table 2:** Interface area and interaction energies.

System	Interface 	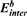	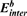
		Arf1-Arno	Arno-GDP
IIo	3278 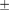 100	−368 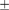 31	−22 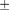 10
IIm1	3070 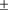 126	−327 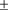 49	22 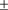 6
IIm2	3034 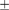 106	−349 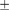 31	13 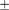 7


 interface area in 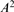.

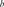
 interaction energy in kcal/mol.

As would be expected for such interfaces, the measured energy of interaction (van der Waals and electrostatic terms) between Arf1 and the GEF is large and negative (Table 2). The proportionality of the energy of interaction and interface area is not expected to be exact; however the interaction energy was seen to be significantly more negative in the Mg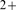-free form of the intermediate complex II than in the other forms with lesser interface areas.

**Figure 4:**
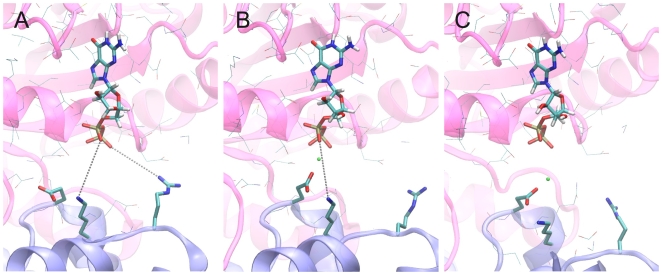
Representative structures for each of the three models of the Arf1-GDP-GEF complex. Shown are sampled MD structures closest in an all-atom rmsd sense (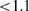 A in all cases) to the ensemble average for each of the three models of the Arf1-GDP-GEF complex. *A:* the Mg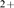-free complex IIo, *B:* complex IIm1 containing Mg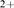, *C:* complex IIm2 containing Mg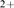 in an alternative position. Arf1 is colored rose, the GEF light blue. In each panel GEF residues Glu156, Lys159, and Arg118 are shown. The Mg ion, when present, is indicated by a green sphere. Lines indicate distances from phosphate oxygens to Lys159 and Arg118 when less than 8 A. Distances are 7.1 and 6.4 A, respectively, in IIo (*A*), 7.3 and 9.8 A in species IIm1 (*B*), and 9.2 and 8.3 A in species IIm2 (*C*). The corresponding averaged distances for the three systems are 7.4 and 7.6 A for IIo, 7.6 and 9.7 A for IIm1, and 8.6 and 9.9 A for IIm2.

**Figure 5:**
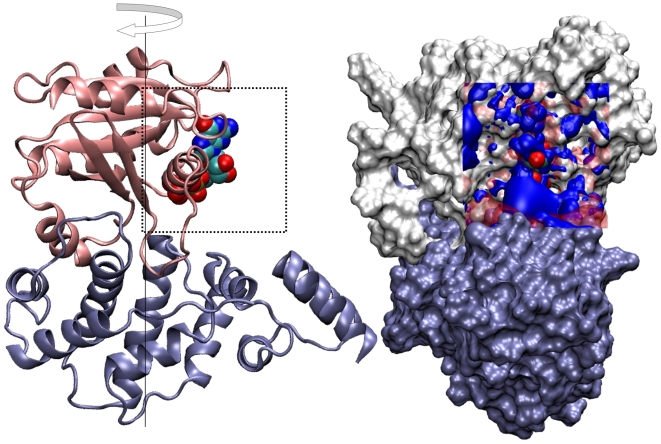
Electrostatic potential differences between intermediate Arf1-GDP-GEF complexes. Arf1 at top and the GEF at bottom. As in the previous Figure, the complex shown at left is rotated by 90 deg about the vertical axis such that the GDP (with phosphates shown in space-filling representation) is facing the viewer. Difference electrostatic potential isosurfaces are shown for the intermediates IIo–IIm2, showing the increased positive potential in the interface region in species IIo. The potential grid for each species was obtained using focusing on the restrained cubic volume shown, centered on atom O3' of the GDP with a grid step of 0.25 A. Only protein and GDP atoms were retained in each calculation for consistency. The 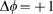 kcal/mol 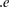 is shown in blue, 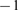 kcal/mol 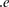 in red, the latter rendered here as a transparent surface.

In addition to the improved protein-protein interaction energy, the GEF also shows significantly stronger interaction with the GDP in the absence of Mg than in its presence– in the latter case the interaction energy is positive (Table 2). A significant component of the improved interaction in the absence of Mg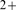 is electrostatic in nature. Figure 4 shows the GDP binding region in representative structures of the three different complexes IIo, IIm1, and IIm2 having the closest correspondence (1.1 A all-atom rmsd) to the ensemble average in each case. The three structures demonstrate significant differences in terms of the proximity of GEF N-terminal subdomain residues to the GDP. The basic residues Lys 159 and Arg 118 can be seen to approach the GDP more closely in the Mg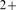-free model IIo compared to IIm1 and IIm2. On the other hand, the catalytic residue Glu156 is more distorted in the absence of Mg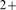, reflecting electrostatic repulsion by the GDP. We calculated the contributions of the protein and GDP to the electrostatic potential for the three models, focusing on the region of the GDP. The results are shown in Figure 5. In this figure the closer approach of positively-charged GEF residues can be seen to lead to a marked augmentation in the positive potential (in blue) at the Arf1-GEF interface near the GDP phosphates. This is consistent with the more favorable GDP-GEF interaction seen in the energy calculations and the electrostatic destabilization of the Mg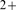 binding site.

### Free Energy Calculations

The differences in interaction energy seen between different models of the pre-dissociation intermediate suggest differences in the GEF and Mg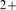 binding affinities. However, binding affinities are a function not only of the interaction energy between partners in the complex but also of the solvation energy compared to the unbound states. In order to estimate free-energy changes for the formation of the different model complexes we used an MM-PBSA approach [25], [30], [31].

Table 3 shows the MM-PBSA results for GEF binding to Arf1-GDP, calculated over 100 snapshots (every 40 ps) of the production MD for the three models of the pre-GDP-dissociation complex (intermediate II). Free-energy changes are given along with the components of the solvation free energy change, 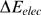 (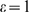) and 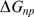, and the interaction energy term 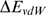. Values of 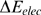 obtained using a protein dielectric constant of 4 are also presented. Calculations performed using two different sets of radii for the Poisson-Boltzmann analyses (see Methods) showed insignificant differences in the final values, so only those obtained using the Charmm radii are reported.

**Table 3:** MM-PBSA analyses of GEF binding*^a^*.

System	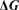	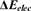	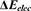	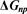	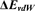
		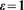	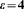		
IIo	−59.7 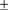 13.1	88.6 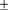 15.8	15.8 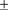 3.5	−14.9 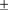 0.6	−133.5 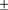 7.0
IIm1	−11.0 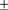 34.2	130.2 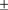 36.2	27.6 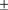 8.2	−14.4 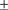 0.7	−126.8 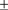 7.5
IIm2	−34.6 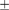 20.1	91.9 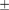 19.7	16.8 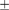 4.7	−14.3 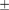 0.6	−112.1 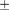 8.1


 all values given in kcal/mol.

The affinity estimates can be interpreted in light of the following schema, which shows the GDP, Mg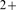, and GEF binding reactions of Arf1:
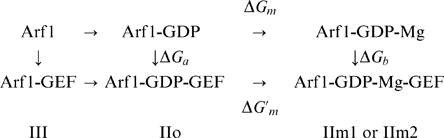



The MM-PBSA calculations correspond to the indicated vertical legs of this schema: 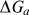, representing GEF binding to form the Mg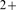-free complex IIo, and 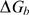, for GEF binding to create either IIm1 or IIm2 containing Mg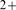. The resulting thermodynamic cycle allows one to express the free energy of Mg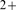 binding to the Arf1-GDP-GEF complex as
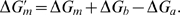
(1)


The strength of Mg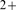 binding to the complex is thus seen to be obtained indirectly from the difference in GEF binding to the Mg-free and the Mg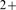-containing forms, in an example of a linkage relation [32]. In contrast to the directly-measured Mg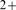 interaction energies shown earlier, the values presented in Table 3 should not be strongly dependent on the Mg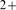 parameterization or polarizability, as they are calculated by measuring differences in protein-protein binding affinities, and not Mg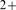 or GDP binding directly.

Comparing the distributions of calculated free-energy changes indicated that the Mg-free Arf1-GDP-GEF complex IIo is significantly (Student's t-test, p = 0.0001) more stable than the Mg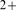-containing complexes IIm1 and IIm2, confirming the reasoning based on the interaction energy and buried surface differences provided above. Application of the linkage relation shows that the Mg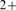 binding affinity is greatly reduced in intermediate complexes IIm1 and IIm2, with the association free energy increasing by 25 kcal/mol and 49 kcal/mol, respectively.

The effect of including limited intrinsic protein flexibility on the electrostatic energy differences, by varying the protein dielectric from 1 to 4, reduced the overall unfavorable contribution of 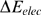 to GEF binding (Table 3) but did not change the ranking of the different modelled species. Indeed, for each term in Table 3, species IIo is favored over the two Mg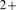-containing species, meaning that the ranking of the GEF affinities for the different species is also independent of changes in the proportionality constant 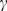 in equation 2 (see Methods).

## Discussion

The small G protein Arf1 has yielded extensive biochemical and crystal structure information and provides an exceptional model for understanding GEF-catalyzed nucleotide exchange in detail. Since the discovery of the Sec7-domain family of GEFs, different mechanisms for the enhancement of nucleotide exchange have been proposed. One of the first suggested that the GEF bound the myristoyl group of Arf directly [33], supposing that accompanying structural changes would result in release of the nucleotide. A subsequent suggestion that closure of the hydrophobic groove produced GDP expulsion [18] was itself later seen to be inconsistent, first with our own normal mode calculations [34] and then more directly with the crystal structures of the BFA-blocked complex (intermediate I) in which the hydrophobic groove was closed but the nucleotide remained in place [6], [19]. This blocked complex has been exploited successfully as a target for structure-based discovery of a new inhibitor of Arf activation [8], highlighting the interest of identifying and structurally characterizing reaction intermediates in drug design.

One point of agreement in mechanistic studies of small G proteins is the importance of destabilizing the GDP in its binding site. Nevertheless, beyond sequestering the Arf1 beta strand 40–50 in the hydrophobic groove of the GEF, a clearly necessary but not sufficient step, it is still not clear how this destabilization takes place. Knowledge of the fate of the Mg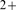 is essential, as the mechanistic consequences of its absence or presence will be clearly quite different for the subsequent steps resulting in GDP dissociation. Even in the absence of the GEF, removal of Mg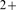 (by addition of EDTA) accelerates nucleotide exchange by a factor of about 20, although this is small compared to the factor of 20,000 due to normal GEF action [23]. Simulation studies of GDP-bound small G proteins, including Arf1, both with and without Mg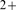 [21], suggested structural consequences that could help explain the necessity for removing this ion in order to destabilize the G-protein-GDP complex. But studies of the early intermediate (I) captured by inhibition with the small molecule by Brefeldin A (BFA), showed the Mg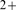 to remain bound to the GDP [6], [19]. Thus the initial interaction of the GEF with Arf1-GDP does not in itself result in Mg unbinding. This led to the suggestion [19] that the GEF ejected both GDP and the Mg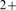 in the next step of the reaction.

The MD simulations and free-energy calculations presented here, performed with native-sequence, exchange-competent components, suggest that intermediate II in the nucleotide exchange reaction is best represented by the Mg-free model IIo of the Arf1-GDP-GEF complex. The Mg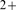-free complex presented a significantly larger protein-protein interface than the Mg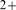-containing versions. The presence of Mg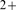, in two alternative placements, prevented the basic residues from the GEF N-terminal subdomain from approaching the GDP binding site as close as they could in the Mg-free complex. The formation of close Arf1-GEF interactions in passing from intermediate I to II would thus play a dual role, first in promoting the rearrangement of the interswitch in Arf1 and, second, in promoting dissociation of the Mg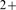. Our theoretical results on Mg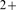 destabilization are consistent with an NMR study [22] that suggested that both Arno mutants E156K and E156A result in abortive Arf1-GDP-GEF complexes accompanied by Mg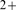 release. Taken together, these results would indicate that the Mg ion is displaced in passing from intermediate conformation I to II in the exchange reaction, and thus that the Mg-free intermediate II is the immediate precursor to GDP ejection. In the “Rho of plants” system, the structure of a predissociation complex [35] showed the Mg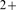 binding site to be occluded by an alanine residue coming from the G-protein itself. Although this is clearly a different mechanism from that suggested here for Arf1, those authors suggested that the dissociation of Mg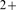 prior to GDP dissociation may be necessary in all G-proteins. It must be cautioned, however, that Mg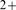 destabilization by the GEF is still only one part of the picture. In EF-Tu, for example, Mg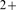 removal increases spontaneous GDP dissociation by up to 300 fold, but its effect on nucleotide dissociation from the EF-Tu-Ts complex is only twofold [36], [37].

In our results, the Mg ion has a clear destabilizing effect on the intermediate II complex and interacts less well than in the inactive Arf1-GDP alone. This presumably results from the mutual repulsion by the divalent Mg ion and several positively charged GEF residues, which, along with the N-terminal of the GEF helix 7, cannot approach their stable positions in the Arf1-GDP-GEF interface. It would be reasonable to conclude that the approach of these positively charged GEF residues to the GDP helps “pay” for the electrostatic repulsion between the GEF Glu156 and the GDP phosphates, and that the presence of the Mg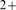 both prevents the approach of this subdomain and annuls the repulsive interaction between the two negatively-charged moieties. In the succeeding steps of the exchange reaction, the approach of GTP-Mg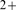 would then act to reverse these effects, promoting the breakdown of the close interactions between Arf1 and the GEF in the nucleotide-free intermediate II.

The structure of the mutationally inactived Arf-GDP-GEF complex 1r8s [6] is the closest pre-GDP-dissociation complex to the nucleotide-free intermediate [15] yet obtained. Nevertheless, the exact nature of the native species approximated by this crystal structure has so far remained unclear. As we have described here, in silico re-integration of the catalytic glutamic finger to create a three-dimensional model of the corresponding native-sequence–and thus exchange-competent–intermediate enabled us to elucidate several important aspects of the critical steps involved in GDP ejection. The recreated intermediate II provides a self-consistent departure point for more detailed mechanistic studies of the GEF-assisted exchange reaction, using theoretical methods appropriate for the study of reaction paths (*e.g.*, reference [38]). Further, the Mg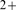-free intermediate II species provides a new pharmaceutical target for potentially modulating Arf1 up-regulation at a critical point in the activation pathway.

### Limitations of the Current Study

As in any modelling and simulation study, certain approximations were necessary in this work. The intermediate species were modelled on the known structure of an inactive intermediate complex. We note that this reconstruction step, involving the modification of a single protein residue, is much milder than that used successfully in other comparative modelling studies, where sequence identity is often in the range of 40–50%. The fixed-charge representation used in the present study is currently the most commonly used MD methodology. However, it is not as realistic as more computationally expensive approaches including effects of atom polarization, which are not yet widely employed in macromolecular simulations. Expected differences from the inclusion of polarization would include reduction in the magnitudes of the calculated protein-Mg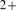 interaction energies shown in Figure 3 for all three systems, as the Mg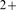 formal charge would be partially compensated by polarization of nearby atoms [39], [40]. On the other hand, the qualitative destabilization of the Mg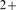 by the GEF seen in the same Figure would be unlikely to be modified by the inclusion of polarizability. In a related vein, and as discussed in a recent study of the Mg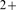-containing EF-Tu system which also employed a fixed-charge parameterization and the Charmm force field [41], there are few highly polarizable protein atoms in the vicinity of the Mg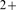. The inclusion of polarization is thus unlikely to significantly affect the indirect measure of Mg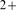 binding from differences in protein-protein binding affinities (Table 3, equation 2). Finally, an additional source of error arises from potentially poorer sampling due to the inclusion of the Mg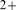. In a study of the double-helical 16S rRNA [42], the inclusion of 24 Mg ions did show differences in detailed structural properties of the nucleic acid when compared to a simulation containing only monovalent ions, which were attributed to sampling inadequacies. However, the overall collective motions of the nucleic acid, as measured by the essential dynamics (principal component analysis) of the macromolecular movements, were very similar. The importance of such potential sampling effects would be difficult to identify unambiguously without much longer simulations.

## Methods

### Comparative Modelling

Studies were based on the pdb entry 1r8s of human Arf1 in complex with a mutant of ARNO, its GEF, in the abortive complex Arf1-GDP-ARNO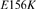 [6]. Corresponding studies of the Arf1-GDP complex were based on the 1hur crystal structure [26]. In the Arf1-GDP-ARNO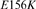 complex, the so-called catalytic GEF (ARNO) residue Glu156 was modelled back into the structure using Modeller [43], using 1r8s as template and allowing modifications to residues within 5 A of the mutated residues. One Mg-free and two Mg-containing models were created. Two different Mg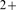 placements were studied: first at the observed position in the Glu156-containing Arf1-GDP-BFA-GEF intermediate 1s9d (after GDP superposition); second at the coordinates of the N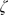 from the Lys substituting Glu156 in the mutant GEF in the 1r8s crystal structure. In each case non-obstructed water molecules around the ion position were brought over along with the Mg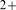 from the corresponding crystal structure. The model with the lowest objective function was chosen for further study in each case.

### Molecular Dynamics Simulations

The molecular simulation program Charmm [44] version 33, using the param27 all-hydrogen parameter set and CMAP terms [45], was used for molecular dynamics calculations and subsequent analyses. Na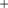 and Cl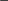 counterions, as well as bound waters, were placed using Solvate [46]. Dynamics trajectories (1 ns equilibration, 5 ns total for each trajectory, 30–40,000 atoms depending on the system) were run in the NPT ensemble (1 atm, 300 K) using periodic boundary conditions and rhombic dodecahedral geometry. Force shift electrostatics and a nonbonded cutoff (12 A) were used in an approach that has proved satisfactory in free-energy decomposition studies of the Arf1-GDP-BFA-Arno complex [47] and other work [48]. As verified by electrostatics calculations (see below), standard ionization states were assigned to all protein residues, while the GDP was assigned a total charge of −3, consistent with the presence of the salt bridge with Lys30 of Arf1. For all models the system was energy minimized using harmonic restraints on the starting heavy atoms about their initial positions; the force constant, initially set at 250 kcal/mol-A, was reduced in a stepwise fashion during successive rounds of minimization until its value fell below 10 kcal/mol-A, and was removed completely before final minimization. Using SHAKE to constrain heavy-atom-hydrogen covalent bonds and a different random seed for each trajectory, the system was then heated to 300 K in 25 degree NVE dynamics steps, during which the stability of the simulation was verified. This was followed by equilibration for 1 ns in the final NPT ensemble before the production phase. Inital tests showed that the use of 1 or 2 fs integration timesteps resulted in simulations of very similar stability, so the 2 fs value was used for all reported runs.

### MM-PBSA Calculations

Free energy changes associated with Arf1-GEF association were estimated by the MM-PBSA approach using snapshot structures taken from the MD simulations of the complexes. In this method the free-energy of each species is taken as the sum of the molecular mechanics energy, a free energy change associated with the transfer of the species to aqueous solution, and an entropy term. For a given species the aqueous solvation contribution can be broken down into the electrostatic work 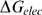 of charging the species in continuum solvent with dielectric 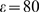, obtained here by solution of the Poisson-Boltzmann equation, and a non-polar surface solvation contribution 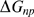 which was estimated by a surface tension, 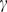, multiplied by the solvent accessible surface 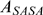. In the snapshot approach, which uses MD simulations of the complex alone, the molecular mechanics term retains only the van der Waals interaction terms between components, calculated with no cutoff. For the reaction 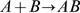, the free-energy difference is then
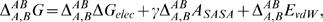
(2)in which entropy terms are assumed to cancel as discussed in other studies [47], [49]. In the present study 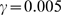 kcal/mol-A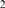 was used [31]. Equation 2 was calculated for 100 MD snapshots obtained at 40 ps intervals from the MD simulations of the Arf1-GEF complex and averaged over each trajectory. Electrostatic energies were calculated using the PBEQ-Solver procedure [50] using an initial grid spacing of 1 A followed by focussing with a grid step of 0.4 A, which was incorporated into an adaptation of the binding energy protocol from the Roux group [thallium.bsd.uchicago.edu/RouxLab]. Electrostatic calculations were carried out using either the Charmm atomic radii or a set of optimized radii determined initially for proteins and augmented for nucleic acids [51], [52]; in the latter case an Mg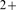 radius of 1.55 A was assigned. Calculations were performed with an ionic strength of 150 mM and protein dielectric constants of 1 or 4 as described in the Results.

### Electrostatics Calculations and pKa Determinations

The most probable ionization states in the complexes at pH 7 were verified using the approach of Antosiewicz et al. (1994) [53], modified in order to allow the inclusion of an additional ionizable phosphate oxygen, for which the model pKa of 6.4 in aqueous solution was used [54]. Electrostatic analyses were made for energy-minimized structures using UHBD [55] to solve the finite-difference Poisson-Boltzmann equation on a cubic grid of length 110 using four-step electrostatic focussing with grid spacings decreasing from 2 to 0.25 A. These calculations employed Charmm atomic radii, an ionic strength of 150 mM, a smoothed molecular surface dielectric-boundary with a 2 A Stern layer, and protein/solvent dielectric constants of 20/80 [54]. Calculated pKa's were consistent with the standard ionization states at pH 7 in all cases, with the exception of the additional ionization center at atom O2B of the GDP. For this group a pKa of 1.7 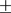 0.7 was obtained, confirming its essentially complete ionization at pH 7 due to the salt bridge formed with Arf1-Lys30. The triply ionized state of the GDP was thus used in all MD simulations.

All graphical molecular representations in this study were generated using VMD [56].
